# A Long-Standing Giant Mandibular Ameloblastoma and its Management with Microvascular Free Fibular Graft: a Case Report

**DOI:** 10.30476/DENTJODS.2020.81805.0

**Published:** 2021-03

**Authors:** Mohammad Saleh Khaghaninejad, Rasoul Gheisari, Hamed Gheibollahi, Saeid Tavanafar, Amirreza Dehghanian, Abbas Jamali

**Affiliations:** 1 Dept. of Oral and Maxillofacial Surgery, School of Dentistry, Shahid Rajaei Acute Care Surgical Hospital, Shiraz University of Medical Sciences, Shiraz, Iran; 2 Postgraduate Student, Dept. of Oral and Maxillofacial Surgery, School of Dentistry, Shahid Rajaei Acute Care Surgical Hospital, Shiraz University of Medical Sciences, Shiraz, Iran; 3 Dept. of Surgical and Clinical Pathology, Molecular Pathology and Cytogenetics Ward, Dept. of Pathology, Shiraz University of Medical Sciences, Shiraz, Iran; 4 Computed Tomography Technician, Shahid Rajaei Acute Care Surgical Hospital, Shiraz University of Medical Sciences, Shiraz, Iran

**Keywords:** Ameloblastoma, Fibula, Mandible

## Abstract

Ameloblastoma is one of the most common benign epithelial odontogenic tumors of jaws. We report a case of long-standing slow-growing giant ameloblastoma involving almost all of mandibular bone. The solid multicystic lesion was excised, and the histopathological examination showed the follicular type of ameloblastoma. Furthermore, the defect was reconstructed with microvascular osteocutaneous free fibular graft.

## Introduction

Ameloblastoma originates from odontogenic epithelium and it is one of the most common benign odontogenic tumors of the jaw. It may develop from a dental lamina, enamel organ, an odontogenic cyst lining, or basal cells layer of the oral mucosa [ 1 - 2 ]. The cause of the tumoral transformation of oral epithelium is still unknown. Although ameloblastoma commonly occurs in the molar-ramus area of the mandible, it can arise anywhere in both jaws. Lesions are mostly asympto-matic and found as a painless expansile jaw growth on routine radiographs from the maxillofacial region [ 1 - 2 ]. It usually grows slowly and can reach a gigantic size if left untreated [ 3 ]. Neural alteration is uncommon even in large lesions. Radical resection recommended in approaching these benign locally aggressive lesions because of the high recurrence rate when surgical enucleation is performed. Long-standing ameloblas-tomas or those with repeat recurrence lesion may transform into ameloblastic carcinoma [ 4 ]. We report a case of massive ameloblastoma involving the mandible which has been left untreated for 20 years. We also described its management with a free fibular graft. The novelty of the present study was the size, long standing duration of the lesion, and the management of this lesion, which would worth mentioning in the literature.

## Case Presentation

**Figure 1:**
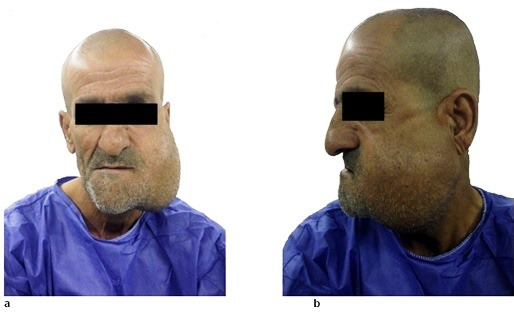
**a:** Preoperative frontal and **b:** lateral view

The patient was asked to sign an informed consent for the surgical procedure and also to use necessary information for reporting this case. A 63 years-old farmer resided in the remote rural area presented with an enormous asymptomatic swelling in the left side of the mandible (Figure 1). The patient had no remarkable medical or family history. He recalls a trauma to face about 33 years ago, recieving a check-up but no treatment was required. Then sometimes later he remembers starting of swelling which has grown slowly until the date he sought treatment. There was no pain, but he reported altered sensation and numbness in the chin area. The facial nerve was intact without any facial palsy. Oral examination reveals very poor oral hygiene with many extracted and retained roots. The swelling was well-limited, non-tender and, extending from the left mandibular condyle and coronoid process to right mandibular body in the 2^nd^ molar region.

**Figure 2:**
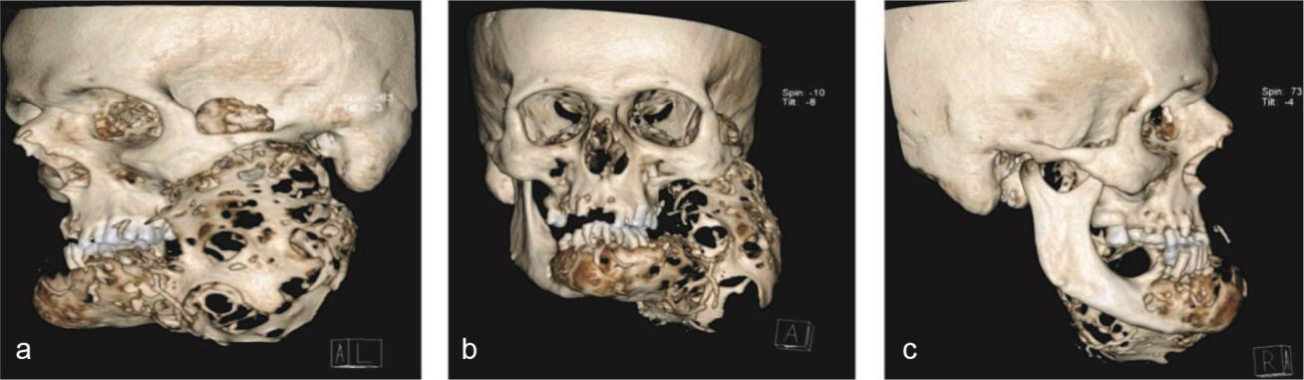
Preoperative computed tomography of the lesion (**a:** Frontal, **b:** Left, **c:** Right)

**Figure 3:**
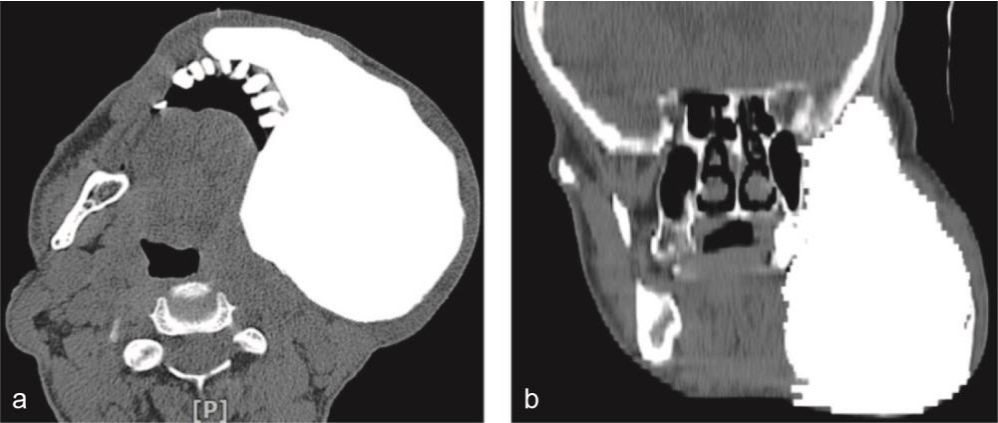
Preoperative computed tomography volume measurement (**a:** cross-section, **b:** coronal)

Computed tomography scan showed a multicystic lesion extending from the left side of the mandible passing over the midline to involve the right 2^nd^ mandibular molar area (Figure 2). The volume of the lesion was 460.65cm3 measured in coronal and axial section of computed tomography (Figure 3). The lesion was extended well beyond zygomatic arch at the level of the zygomaticofrontal suture. There was no maxillary bone or orbital wall involvement with the lesion. Blood examination showed a normal white blood cell count (6.8 ×10^3^/uL), serum calcium level (8.2 mg/dL), and the alkaline phosphatase (ALP: 139 IU/L) level. 

A biopsy performed under local anesthesia, and the histopathologic diagnosis was follicular ameloblastoma. The tumor resected with safe margins under general anaesthesia. A preauricular incision on the left side extended to the right submandibular area, and the mandible was resected, leaving right ramus of the mandible. The facial nerve branches were explored, exposed, and preserved. The treatment was subtotal segmental mandibulectomy with a reconstruction plate- the treatment completed after six months with vascularised osteocutanoues free fibular graft. The histopathologic examination confirmed the diagnosis of a follicular type of ameloblastoma.

### Microscopic and immunohistochemical examination of the specimen

**Figure 4:**
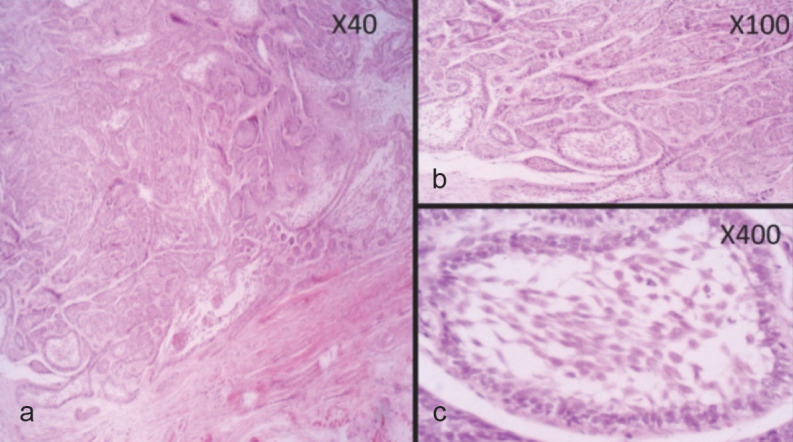
Histopathology evaluation of section shows odontogenic epithelial islands composed of peripheral palisading columnar cells at basal layer in which hyperchromatic cells show reverse polarization away from basement membrane (A,B =40× and 100×, H&E), stellate reticulum-like cells in the centre of nests (C, 400×, H&E) and suprabasal cells composed of loosely arranged angular cells. No dentin or enamel formation is found. No stromal invasion is identified

The lesion was composed of large cystic areas with no necrotic portions. Solid ameloblastic -like cells proliferation with cystic areas suggests the follicular type of ameloblastoma. Microscopic examination at higher magnification showed randomly arranged stellated cells at the center of the nests and columnar epithelial cells at the borders. The columnar cells aligned in palisaded arrangement with reverse polarization similar to inner enamel cells. No malignant features observed (Figure 4).

## Discussion

Ameloblastoma arises from oral ectoderm and has an aggressive and benign progression. It is one of the most common epithelial odontogenic tumors of jaws [ 5 ]. It has a very high recurrence rate (up to 50%) if treated inadequately and in less than 1% shows the malignant transformation. Mandibular ramus is the most common site of odontogenic epithelial tumours and ameloblastoma takes a slow-growing course, but on occasion, it reaches a gigantic size. There is no consensus on the definition of giant ameloblastoma. Some authors suggested any lesion with an axis of 5cm or more to be considered as giant ameloblastoma [ 6 ], while others recommend assessing the marginal invasion of the lesion and its volume without numerical limits [ 7 ]. We presented a male patient in his 6th decade of life with a large lesion of the lower left side of the face. The patient had neglected the lesion for an economic reason and did not seek treatment until he could not open his mouth for proper eating. While previous reports showed higher frequency in male [ 8 ], others found that lesion affects young women [ 9 ]. The age of the patient in the present study was higher due to patient neglecting the necessary treatment, although patients recall a trauma to face about 30 years ago (in his 30s), which consistent with other reports. The present lesion showed a radiographically intricate multicystic pattern which is considered frequent in the ameloblastic lesion [ 10 ]. Similar to the present case, the majority of giant ameloblastomas are highly vascular which is related to their active tumoral proliferation, and may require preceded embolization [ 11 ]. Fortunately, our case was managed without embolization. Detection of the lesion is usually accidental and during a radiographic examination of the jaw for another reason. Asymptomatic progression sometimes delays earlier treatment, although some patients experience signs and symptoms such as swelling, pain, altered sensation, dental malocclusion, and facial deformity [ 12 ].

Computed tomography can reveal a well-defined uni or multicellular, cystic or solid lesion with or without a cortical wall.

Three variants of ameloblastoma with distinct clinic al and radiographical features require special considerations because of the difference in their treatment approaches and prognosis. Multicystic or solid ameloblastoma accounts for 86% of all types, which is the most common type of ameloblastoma, and our case also falls in this category. Unicyctic type of ameloblastoma accounts of 13% and the remaining 1% of all ameloblastoma are the peripheral type. Follicular and plexiform types are the most common histopathologic subtypes of conventional solid or multicystic ameloblastomas. Other subtypes are acanthomatous, basal cell, desmoplastic, and granular cell type. The present case showed a follicular subtype, and there was no nerve alteration of the trigeminal or facial nerve associated with this case. Although this case had been growing over twenty years, no elevation in serum calcium was observed. In the present case, we measured the volume instead of giving length, width, and height of the lesion. The volume measurement might provide a better estimation of the size of the lesion because of the multidirectional growth, which is difficult to measure. Ameloblastoma recurrence is frequent, and the solid/multicystic form have more local aggression and recurs significantly more frequently if not excised completely, while unicystic ameloblastoma has less aggressive behavior [ 13 ]. Thus, in our case, it was preferred by the surgeon to wait before reconstruction of the defect. After ensuring no recurrence, the reconstruction was performed.

There is a lack of agreement toward the most appropriate treatment modality between clinicians. Those who advocate conservative treatments such as curettage, enucleation, and cryotherapy presume that these lesions are mostly benign although locally invasive. These conservative approaches have low morbidity than radical modalities such as marginal, segmental, and hemim-andibulectomy. Many authors recommend enucleation with periosteal preservation, especially in young children which is essential for bone regeneration [ 14 ]. Advocators of radical treatments believe that curettage and enucleation of ameloblastoma lead to a high recurrence rate which has been reported up to 55-90% in the literature [ 15 ]. We also believe that radical surgical excision with a margin of 2cm of healthy bone is the best approach in treating these cases.

**Figure 5:**
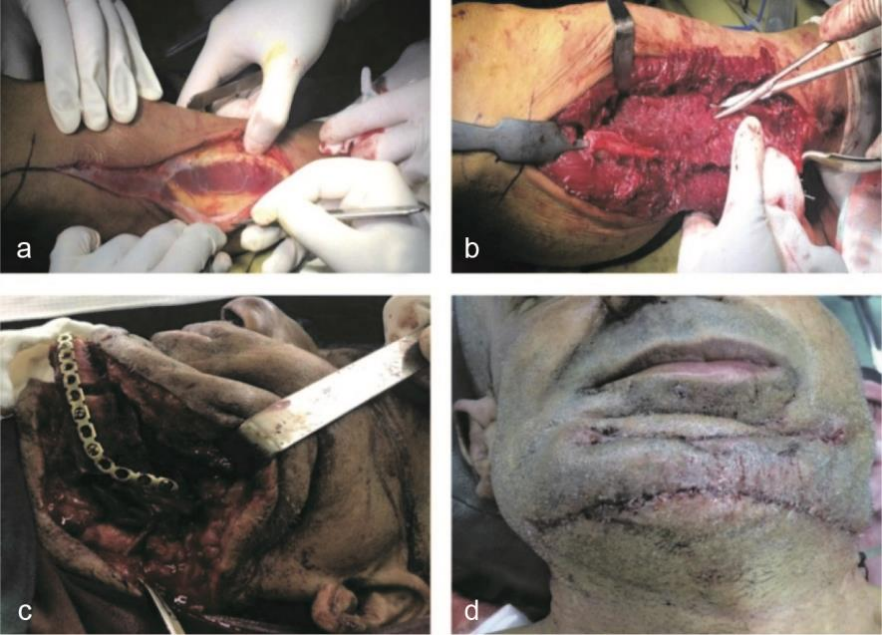
Free fibular graft harvest and mandibular reconstruction (A: Flap design, B: Graft harvest, C: Microvascular anastomosis and graft placement, D: final result)

The bony margin represents a distance from the radiographical margin, which is considered to be disease-free, and it is oncologically safe to perform osteotomies. The structural pattern of ameloblastoma in cancellous bone is such that the border of the tumor might lie well beyond the visible macroscopic surface and radiographic boundaries [ 16 ]. On account of the large size of the lesion in our case and significant bone defect postoperatively, we have used free fibular flap for mandibular reconstruction (Figure 5).

Some of many advantages of the free fibular graft are a capability of a large amount of bone (up to 25cm), soft tissue, and if necessary, skin transfer(up to 25cm in length and 5cm in width) with only one donor site. The fibula blood supply is also both intraosseous and segmental, which allows multiple oste-otomies to shape the bone similar to the mandible [ 15 ]. Patient and his gaurdians singined a written informed consent for reporting this case.

## Conclusion

Microvascular free fibular graft shows a predictable result although it is a technically demanding procedure and experience plays a vital role in reconstructing jaw defects with this method.
